# Water Sorption, Water Solubility, and Rheological Properties of Resin-Based Dental Composites Incorporating Immobilizable Eugenol-Derivative Monomer

**DOI:** 10.3390/polym14030366

**Published:** 2022-01-18

**Authors:** Ali Alrahlah, Abdel-Basit Al-Odayni, Waseem Sharaf Saeed, Abdullah Al-Kahtani, Fahad M. Alkhtani, Nassr S. Al-Maflehi

**Affiliations:** 1 Restorative Dental Sciences Department, College of Dentistry, King Saud University, Riyadh 11545, Saudi Arabia; 2 Engineer Abdullah Bugshan Research Chair for Dental and Oral Rehabilitation, College of Dentistry, King Saud University, Riyadh 11545, Saudi Arabia; wsaeed@ksu.edu.sa; 3 Chemistry Department, College of Science, King Saud University, Riyadh 11451, Saudi Arabia; akahtani@ksu.edu.sa; 4 Department of Prosthodontics, College of Dentistry, Prince Sattam Bin Abdulaziz University, Alkharj 13 11924, Saudi Arabia; f.alkhtani@psau.edu.sa; 5 Department of Periodontics and Community Dentistry, College of Dentistry, King Saud University, Riyadh 11545, Saudi Arabia; nassr@ksu.edu.sa

**Keywords:** polymerizable eugenol, dental composite, water sorption, water solubility, rheology

## Abstract

The present study aimed to evaluate the properties of new dental formulations incorporating a new polymerizable-derivative of eugenol (EgGMA). The experimental composites were prepared (by weight) with 35% resin-based matrix (1:1, bisphenol A-glycidyl methacrylate/triethylene glycol dimethacrylate) and 65% reinforcing materials (4:3, hydroxyapatite/zirconium oxide). A portion of 0.0, 2.5, and 5.0% of the resins with respect to the total composite was replaced by EgGMA monomer to obtain TBEg0, TBEg2.5, and TBEg5, respectively. The complex viscosity (at 25 and 37 °C), degree of conversion (DC), and water sorption (W_SP_) and water solubility (W_SL_) (3 cycles of sorption-desorption process) were investigated. Data were statistically analyzed using one-way and Tukey post-hoc tests. The results revealed a viscosity reduction with shear-thinning behavior as the EgGMA amount and temperature increased. The average complex viscosities at a lower frequency (ω = 1.0 rad/s) and at 25 °C were 234.7 ± 13.4, 86.4 ± 16.5, and 57.3 ± 17.1 (kPa·s) for TBEg0, TBEg2.5, and TBEg5, respectively. The inclusion of EgGMA led to a lower DC and W_SP_ but higher W_SL_, compared to those of the reference (TBEg0). However, no significant differences between TBEg2.5 and control were detected (*p* > 0.05). Therefore, the incorporation of EgGMA in a low quantity, e.g., up to 8.45 mol% of resins, within the matrix may enhance the composite’s performance, including handling and solubility properties without any apparent effect on DC and water sorption, making it a promising monomeric biomaterial for various applications including restorative dentistry.

## 1. Introduction

In the last few years, resin-based composites incorporating immobilized eugenol (Eg) derivatives have received considerable attention as a promising enhancer of restorative dental materials, particularly for intracanal post cementation and core build-up restoration [1,2,3]. The target is to benefit the advantage of Eg moiety as a potent antimicrobial, endow dental restorative materials with sustained antibacterial activity to promote long-term performance [4,5], thus reducing the risk of reinfection [6]. Additionally, the potential effects of additives on the physicochemical, mechanical, and some other properties of resin composites are of great importance to researchers and have to be assessed as well. Though the promising properties of polymerizable Eg-based monomers [1,7], literature screening revealed only a few derivatives that were synthesized and researched for application as dental and orthopedic materials [8,9].

Eugenyl-2-hydroxypropyl methacrylate (EgGMA) is a methacrylate-based derivative of eugenol which is recently synthesized and its reactivity in reference to methyl methacrylate and biocompatibility within resin composites was assessed [9,10]. The enhanced cell viability of resin-based composites in the presence of EgGMA is a promising feature that encourages further investigation of their various properties as a biomaterial. EgGMA is a low molecular weight (292.33 g/mol) monomer produced via activated condensation reaction of eugenol with glycidyl methacrylate; thus certainly its viscosity is undoubtedly more inferior than the traditional base monomers used in resin-based composites such as bisphenol A-glycidyl methacrylate (BisGMA) and urethane dimethacrylate (UDMA) but higher than the common comonomer triethylene glycol dimethacrylate (TEGDMA). Besides its immobilizability within the base matrix of restorative dental composite, EgGMA may preserve the desirable properties of free Eg molecule [11], thus supporting its application as a potential biomaterial. Moreover, the allylic double bond can, to some extent, be involved in the polymerization reaction, thus increasing the degree of crosslinking [7,8,12], the property that is closely pertinent to mechanical as well as to most of the physicochemical properties, including sorption and solubility of the composite.

It is widely known that Eg has beneficially been implicated in various applications and for several purposes [13,14,15]. Since ancient times, it has been used as an antimicrobial and antiseptic agent. Presently, Eg is admitted as a nutraceutical and pharmaceutical regent, with benefits as an anesthetic, antioxidant, antimicrobial, anti-inflammatory, anti-carcinogenic, neuroprotective, hypolipidemic, and anti-diabetic [16,17] agent. However, its incorporation in dental resin composites is not recommended due to its inhibitory impact on the degree of conversion (DC), which may affect other valuable properties of the composite. Furthermore, its pungent odor and volatility are another reason for the undesirability of its usage in dental composites. Eg in combination with zinc oxide (ZnO_2_) is a common luting and temporary restorative material, but its low strength and high oral solubility prevent its application as permanent restoratives [17,18,19], thus its polymerizable derivatives may bring solutions [1,2,3,13]. In this regard, Almaroof et al. [1] have investigated various properties of dental composites incorporating eugenyl methacrylate (EgMA) for post and core build-up restoration, reporting EgMA concentration-dependent properties with reduced values of DC and curing depth with EgMA increase. Rojo et al. [8] reported enhanced cement properties of polymerizable Eg-derivatives including mechanical and bactericidal that are attributed to the possible crosslinking driven by allylic group participation. Additionally, physicochemical and mechanical analysis of restorative composites incorporating EgMA and EEgMA have shown a relatively lower DC and water sorption, and moderately comparable water solubility and mechanical properties compared with those of control which has no immobilizable eugenyl moiety.

In the present article, immobilize methacrylate-based derivative of eugenol (EgGMA) was incorporated as 2.5 and 5 wt% in experimental dental composites consisting of 35 wt% base matrix (BisGMA and TEGDMA) and 65 wt% reinforcing model filler (HA and ZrO_2_). The rheological isothermal properties at 25 and 37 °C, DC, as well as the water sorption (W_SP_) and solubility (W_SL_) of model composites, were evaluated for three cycles of the adsorption-desorption process. Then, data were statistically evaluated in reference to EgGMA-free composites as a control. It was hypothesized that (1) the incorporation of EgGMA monomer as 2.5 and 5 wt% will not significantly affect the complex viscosity, DC, W_SP_, and W_SL_ of the resin-based composite, (2) the complex viscosity of each composite would not differ significantly at 25 and 37 °C, and (3) there is no significant differences in W_SP_ and W_SL_ values for three cycles of sorption-desorption processes.

## 2. Experiments

### 2.1. Chemistry

The base monomers BisGMA (>98%) and TEGDMA (>95%), light-initiator camphorquinone (CQ, 97%), co-initiator 2-(dimethylamino)ethyl methacrylate (DMAEMA; 98%)), organosilane coupling agent (3-(trimethoxysilyl)propyl methacrylate (γ-MPS, 98%), filler (HA, ≥97% and ZrO_2_, 99%), synthesis reagents eugenol (Eg, 98.5%) and glycidyl methacrylate (GMA, 98%), and the free radical polymerization inhibitor hydroquinone (HQ, >99%) were purchased from Sigma-Aldrich (Taufkirchen, Germany). Triphenylphosphine (Ph_3_P, 99%) used for EgGMA synthesis catalysis was procured from Cica-reagent (Kanto Chemical, Tokyo, Japan). The solvents used, ethyl acetate (EA, 99.5%) and *n*-hexane (*n*-Hx, 95%) were obtained from Fisher Scientific (Loughborough, England, UK).

Details for EgGMA synthesis are described elsewhere [9] and are summarized below. To equimolar of Eg and GMA, 0.5 wt% and 0.1 wt%, respectively, of HQ and Ph_3_P for the total weight of reactants were added. The reaction was refluxed at 120 °C for 2 h under a nitrogen atmosphere. Thin-layer chromatography (TLC, aluminum plate, silica as stationary phase, and 7:3 EA/*n*-Hx as mobile phase) was used for monitoring the reaction completion and further for purity assessment. The product was purified using silica-gel (60 mesh) column chromatography using the same mobile phase as in TLC. The solvent was removed using a rotary evaporator (Buchi, R-210, Boston LabCo, Boston, MA, USA) and vacuum-dried until a constant weight was achieved. The obtained light-yellow oily monomer of about 66% yield was stored in a refrigerator until use.

Reinforcing materials were modified as below. Zirconia (ZrO_2_, <2 μm particle size) was silanized using a previously described method [20]. Briefly, γ-MPS (0.6 g; 2 wt% with respect to the ZrO_2_) was hydrolyzed in 100 mL acetone with stirring for 2 h. Following, 30 g of ZrO_2_ powder was added and left to stir overnight at room temperature. The next day, the suspension was filtered, washed with acetone, and dried at 100 °C for 3 h. HA powder (<5 μm particle size, surface area ≥ 100 m^2^/g) was modified with γ-MPS following a procedure described elsewhere [21]. Typically, a solution of γ-MPS (5 wt% with respect to HA) in 90 vol% ethanolic aqueous solution was prepared, then the pH was adjusted to 4 by drops of acetic acid. After 90 min of stirring, HA was added in batch, thoroughly dispersed, sonicated for 10 min, and left to stir overnight at room temperature. Finally, the as-obtained product was filtered, washed with ethanol, and dried at 60 °C overnight.

### 2.2. Preparation of TBEg Experimental Composites

EgGMA was incorporated in the model composites at 0, 2.5, and 5 wt% [1] as given in Table 1. Each composite (TBEg0, TBEg2.5, and TBEg5) consists of 35 wt% TBEg (TBEg stand for TEGDMA, BisGMA, and EgGMA), 65 wt% reinforcing materials (silanized HA and silanized ZrO_2_), and an initiator system (0.5 wt% CQ and 1.0 wt% DMAEMA with respect to the total monomers). Initially, a mixture of BisGMA and TEGDMA (1:1 by mass), representing the resin matrix, was prepared and, after that, partially replaced at 0.0, 2.5, and 5.0 wt% by EgGMA monomer. CQ was dissolved in this resin matrix and, subsequently, the fillers were added. The contents were manually mixed using a stainless-steel spatula, followed by mechanical mixing using a dual asymmetric centrifugal mixing system (Speed Mixer TM DAC 150 FVZ, Hauschild and Co., Hamm, Germany) four times (for 1 min each, with 2 min rest in between) at 2500 rpm. The obtained pastes were molded as specified for each test and light-cured using a LED curing light unit (Elipar S10, 3 M ESPE, St. Paul, MN, USA) for 40 s, as detailed below.

### 2.3. Water Sorption and Solubility Tests

Disc-shaped specimens (*n* = 5), 15 mm in diameter and 2 mm in thickness, were prepared in stainless-steel molds and photo-polymerized from both sides in four overlapped areas (40 s each) using a 10-mm-diameter-tip curing unit [22]. To calculate the water sorption (W_SP_) capacity and solubility (W_SL_), the prepared discs were first dried in a desiccator containing dry calcium chloride maintained at 37 ± 1 °C for 24 h, transferred into another desiccator maintained at room temperature 24 ± 1 °C for about 1 h and measured for their weight. The drying process was repeated until constant weight (m_1_) was obtained with an accuracy of 0.1 mg. Next, the discs were immersed in 15 mL distilled water and kept at 37 ± 1 °C for the water uptake test. After 24 h, the samples were carefully removed from the water, swabbed, weighed, and returned to the water. The process was repeated every 24 h until constant weight (m_2_) was achieved. Finally, the specimens were removed from the water and dried at 37 ± 1 °C until constant weight (m_3_). The sorption-desorption process was repeated for three cycles. Data were reported as the average of five replicate. W_SP_ and W_SL_ were calculated using Equations (1) and (2).

(1) $$W_{SP}\left(\%\right)=\left(\frac{m_{2}-m_{1}}{m_{1}}\right)\times 100$$

(2) $$W_{SL}\left(\%\right)=\left(\frac{m_{1}-m_{3}}{m_{1}}\right)\times 100$$

### 2.4. Degree of Conversion

The conversion of polymerizable vinylic (C=C) bonds into single bonds was spectroscopically monitored using the well-known Fourier transform infrared (FTIR) method for the aromatic C=C band as one applicable internal standard. The specimens were fabricated in a disc-shaped stainless-steel mold (5 mm × 2 mm, *n* = 5), and their attenuated total reflection FTIR (ATR-FTIR) spectra were measured (uncured) using a Nicolet iS10 FTIR spectrometer from Thermo Scientific (Madison, WI, USA) equipped with an ATR diamond crystal accessory, in the transmittance mode, at 32 scans per spectrum and a resolution of 4 cm^−1^. Subsequently, the specimens were covered with plastic strips followed by a microscope glass slide, irradiated for 40 s from both sides, and their ATR-FTIR spectra were recorded (cured). Peaks of aromatic and aliphatic double bonds at 1608 and 1638 cm^−1^, respectively, were selected for DC calculation. Thus, the absorption bands were integrated, and the obtained areas were compared as given in Equation (3). Knowing that peak area represents bond mole fraction in the molecule, however, this is true for bonds with similar absorptivity and only from the identical spectra.

(3) $$\mathrm{DC}\;\left(\%\right)=\left[1-\frac{\left(\frac{A_{1638}}{A_{1608}}\right)\mathrm{cured}}{\left(\frac{A_{1638}}{A_{1608}}\right)\mathrm{uncured}}\right]\times 100$$

where A_1638_ and A_1608_ represent the areas of the aliphatic and aromatic bonds at 1638 and 1608 cm^−1^, respectively.

### 2.5. Rheological Measurement

The rheological properties, including dynamic viscoelastic behavior of the freshly prepared uncured experimental composites, were determined in an MCR 72 rheometer (Anton Paar, Graz, Austria) at two measuring temperatures (25 and 37 °C), over a frequency sweep of 0.1–100 (ω, rad/s) in the oscillation mode using a parallel plate of 25 mm, specimen measuring gap of 5 mm, and Peltier plate-temperature control (±0.1) (*n* = 3). Data for complex viscosities (η*, kPa·s) and loss factor (Tan δ) vs. angular frequencies were replotted on the logarithmic measuring scale, and η* at 1.0 rad/s was tabulated for comparison.

### 2.6. Statistical Analysis

Data were statically analyzed using SPSS 21 (IBM Corp., Armonk, NY, USA). Results were presented as mean ± standard deviation (SD) or standard error of the mean (SeM). One-way analysis of variance (ANOVA) followed by Tukey post-hoc and Bonferroni multiple comparison tests were used to analyze the significant differences of W_SP_, W_SL_, complex viscosity, and DC between, respectively, materials (groups, TBEgs) and within the examined property such as temperature-based complex viscosity and repeated sorption-desorption process (3 cycles), respectively. A *p*-value < 0.05 was considered significant.

## 3. Results and Discussion

### 3.1. Rheological Analysis of Uncured Composites

**Figure 1:**
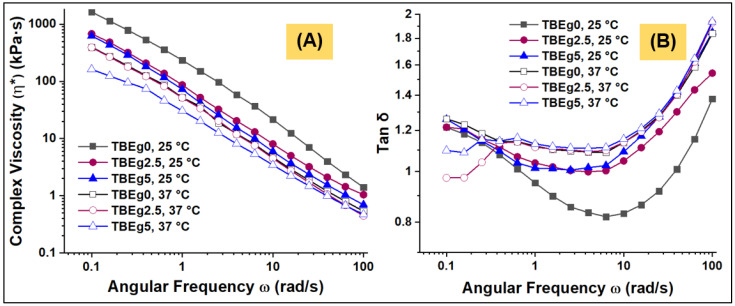
(**A**) complex viscosities (η*, kPa·s) and (**B**) loss factors (tan δ) vs. angular frequency (rad/s) of the model resin-based composites (TBEg0, TBEg2.5, and TBEg5) at 25 and 37 °C.

The viscoelastic behavior of the prepared composites (TBEg0, TBEg2.5, and TBEg5) was evaluated using a rheometry technique at statics temperatures of 25 and 37 °C (*n* = 3). Figure 1 shows the complex viscosity (η*, kPa·s) and loss factor (tan δ) of uncured specimens recorded over the angular frequency (ω) range of 0.1 and 100 rad/s. The data of η* presented at 1.0 rad/s are given in Table 2. As can be seen, all composites were viscoelastic, with thinning characteristics; therefore, as shear frequency increased, the η* value decreased [23]. Additionally, as the EgGMA fraction increased, the η* value also decreased. This is due to the low-molecular-weight and viscosity of EgGMA. Hence, EgGMA addition into resin-based dental composites would enhance the flow properties by reducing the overall viscosities of the composites [24]. At 25 °C, the η* values of both TBEg2.5 and TBEg5 differ significantly from that of the reference (TBEg0) (*p* < 0.05), therefore, the first hypothesis regarding complex viscosity was rejected. However, the difference between TBEg0 and TBEg2.5 at 37 °C was insignificant but both significantly differ from that of TBEg5. This may indicate that the structure-property of composite is not much affected by the addition of a low fraction of TBEg, i.e., ≤2.5 wt% (TBEg2.5 contains 7.14 wt% of matrix fraction, but a 2.5 wt% with respect to the total composite), particularly at a higher temperature. For each composite, the η* was significantly higher at 25 °C than that at 37 °C. Thus, the second hypothesis was rejected. Other tested properties, including W_SP_, W_SP_, and DC, could support this argument. Generally, the high complex viscosity of TBEgs is a result of several mobility restrictions associated with components structures. The organic matrix is rich with hydroxyl groups involved, particularly in the BisGMA structure (with a molecular weight of 512 g/mol and 2 OH per mol), leading to a solid intermolecular interaction, thus reducing mobility [25]. Other forces such as pi-pi interaction caused by aromatic rings and ionic attractions with inorganic parts may participate in the high viscosity of composites. It is clear that the addition of 2.5 wt% EgGMA to a conventional matrix containing, besides TEGDMA, BisGMA, has led to a noticeable decrease in complex viscosity primarily due to the reduction and interruption of the number of possible hydrogen bonding within the matrix.

Furthermore, the thinning behavior could be attributed to the disruption of intermolecular interaction between resin matrix components, which is essentially driven by hydrogen bonding and pi-pi interactions, resulting from shear rate and temperature increase [26]. Compared to TBEg0 (EgGMA-free) composite, the addition of 2.5 and 5 wt% EgGMA has resulted in a reduced η*, as reported for 1.0 rad/s in Table 2, those are from 234.70 to 86.44 and 72.34 at 25 °C, and from 52.01 to 51.81 and 30.59 (kPa·s) at 37 °C; the recorded values of η* at 0.1 rad/s as the lowest ω terminal were about 1634, 676 and 571 at 25 C, and 395, 391 and 161 (kPa·s), for TBEg0, TBEg2.5, and TBEg5, respectively.

Loss factor (tan δ), defined as the ratio between loss modulus (G″, the viscous part or energy loss) and storage modulus (G′, the elastic part or energy stored), is a measure of damping in the material, providing information on viscoelasticity tendency of the material at the applied condition [27,28]. Thus, tan δ greater than unity indicates domination of viscous component. As can be seen in Figure 1B, the loss factor (tan δ) of TBEg0 was the lowest compared to TBEg2.5 and TBEg5. With a temperature increase from 25 °C to 37 °C, tan δ was also increased. At all conditions, the values of the tan δ of tested samples were above one, indicating a predominantly gel-like viscoelastic behavior. The frequency dependency of loss factor showed almost similar profiles of tan δ, which could be defined as three regions: (1) from 0.1–1 rad/s, the tan δ slightly reduced as frequency increased, particularly at 25 °C, the case that is possibly a result of rearrangement of the components, including organic monomers and inorganic layers, facilitated by the low shear rate; (2) from 1–10 rad/s, a region of frequency-independent tan δ in which a plateau profile is observed; however, the tan δ of TBEg0 at 25 °C continue decreased supporting the assumption that the incorporation of EgGMA increases the liquid-like behavior of the composites; (3) from 10–100 rad/s, the increase in tan δ is more pronounced, and all materials have the same profile. The last observation may result from disruption of the interconnecting forces such as ionic and hydrogen bonding between the composite components. This case was also obtained at a higher temperature.

### 3.2. Degree of Conversion, DC

The degree of vinyl double-bond conversion (DC) upon curing TBEg composites as assessed using the FTIR method is given in Table 2. The change in the peak area of the polymerizable aliphatic vinyl C=C, detected at 1638 cm^−1^, with respect to the internal standard aromatic C=C at 1608 cm^−1^, was calculated before and after specimen irradiation (*n* = 3) (Equation (3)). The data obtained indicated a reduced DC with the addition of EgGMA, from 68.75% of TBEg0 to 66.49% and 58.03% for TBEg2.5 and TBEg5, respectively. However, no significant effect of EgGMA addition up to 8.45 mol% of the monomeric components (TBEg2.5), thus the first hypothesis of DC was partially rejected. Such reduction may be due to the reactivity difference between various vinylic groups existing in the resin composites (2 moles in both TEGDMA and BisGMA and 1 mole in EgGMA) and some retained activity inhibitory effect of the Eg moiety. Furthermore, it is reported that the allylic group of EgGMA can, to some extent, participate in the crosslinking reactions, but its reactivity is less than vinylic and can be involved only in post-polymerization events, leading to DC development after 24 h [2,29]. Meanwhile, the apparent inhibitory effect of EgGMA on resin composite at higher concentration (TBEg5), the obtained DC are still above the reported acceptable limit for clinical use (>55%) [11,29,30].

### 3.3. Water Sorption and Water Solubility

**Figure 2:**
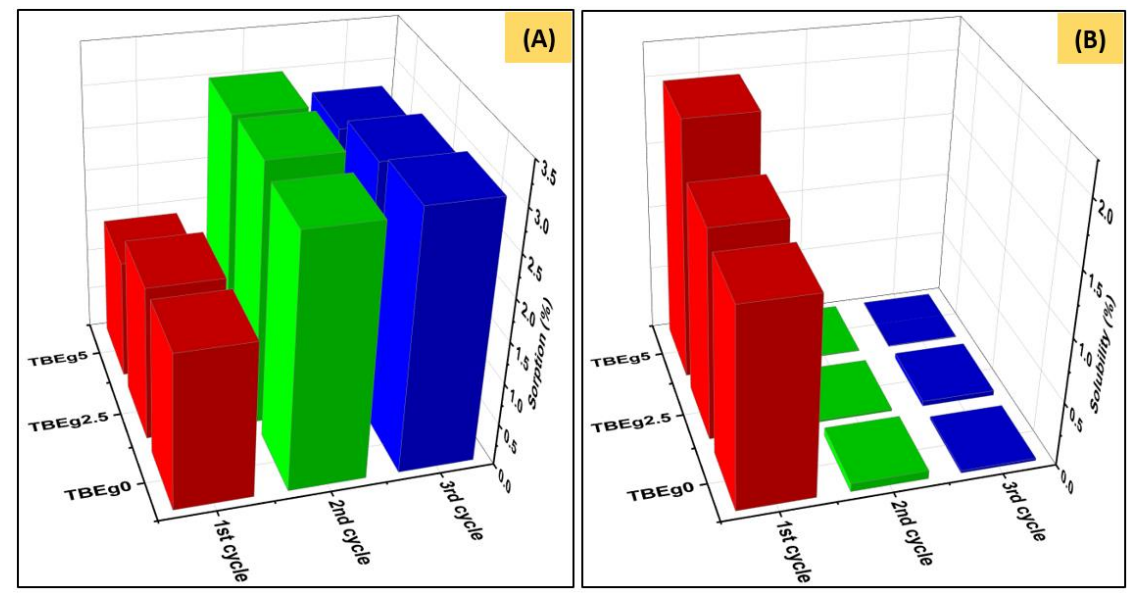
Water sorption (**A**) and water solubility (**B**) of TBEg0, TBEg2.5, and TBEg5 after three cycles of sorption-desorption processes.

The water sorption (W_SP_) and solubility (W_SL_) of the studied model composites were quantified for three cycles of repeated sorption-desorption processes as summarized in Table 2. The results indicate that, as EgGMA amount in the composite increased, the amount of W_SP_ decreases, whereas W_SL_ increases (Figure 2). The change in both W_SP_ and W_SL_ between TBEg0 and TBEg2.5 was insignificant (*p* > 0.05), while became statically significant (*p* < 0.05) as EgGMA content exceeded 8.45 mol% of the organic matrix (e.g., for TBEg5) and, therefore, the first hypothesis regarding W_SP_ and W_SL_ was partially rejected. The decrease in W_SP_ is probably due to a reduction in overall resin hydrophilicity compared with the control (TBEg0) [31]. Furthermore, the BisGMA is a di-OH monomer while EgGMA is mono. Consequently, the mole fraction of BisGMA was reduced from 64.16 in TBEg0 to 58.74 and 53.47 mol% in TBEg2.5 and TBEg5, respectively, the OH mol% was less by half of the replaced BisGMA mole fraction. On the other hand, the increase in W_SL_ among TBEg0-o-TBEg5 goes with the reduced DC, suggesting lesser effective participation of EgGMA moieties in the polymerization, leaving some leachable molecules/oligomers un-immobilized and ready to release, e.g., into the experimental fluid (water) [32,33]. According to the literature, W_SL_ is a poor predictor of monomer DC [34]. Compared with the first cycle of the sorption-desorption process, the sorption is significantly higher in the 2nd cycle, suggesting rejection of the third hypothesis; however, the latter insignificantly differ from that calculated in the 3rd cycle, indicating no more water sorption after cycle two. In contrast, WSL reached the maxima in the first cycle, with no significant release of leachable species in the 2nd and 3rd cycles. Such behavior demonstrates the total elution of the unreacted small molecules in the first cycle. Furthermore, the absorbed water molecules in the first cycle may play an essential role in resin swelling, plasticization, and catastrophic degradation, leading to the insignificant change observed for the next cycles.

Overall, the results of W_SL_ tests revealed slightly higher-solubility (leachable) materials of EgGMA-containing TBEg composites mostly due to incomplete polymerization (i.e., decrease in DC) of the resin matrix; however, the reduced W_SL_ is one advantage of incorporation of EgGMA within the dental composites. Assuming that the amounts of the leachable materials are proportional to the component ratio in the composite, leachable amounts for BisGMA and TEGDMA monomers will decrease, whereas that for EgGMA will increase (EgGMA constitutes about 7 and 14 wt% of the total resin mixtures in the experimental composites, TBEg2.5 and TBEg5, respectively). Although the antioxidant activity of Eg derivatives is less than that of free eugenol molecules, its effectiveness, to some extent, was reported to be retained, functioning through the allylic group [35]; however, the delayed contribution of the allylic group may be another cause of higher solubility at the early stage of sorption process, the first cycle.

Generally, the success rate, integrity and longevity of a composite restoration depend on several variables that may be difficult for the operator to control [36]. Of these factors are the restorative composite, the caries status, treatment procedure, curing condition, patient preferences and dentist decision. As the composite-related and polymerization-affecting factors are important, considerable effort has been devoted by researchers and industry to modify resin restorative materials to improve composite physicochemical properties, handling characteristics, mechanical and clinical performance [36,37]. In this regard, EgGMA was developed as a potential additive monomer for resin restoratives and its initial assessment has shown biocompatibility enhancement and a comparable degree of polymerization to that of EgGMA-free conventional model resin composite. Such properties were evidenced as EgGMA dose-dependent and have shown not to exceed 8 mol% per resin for desired performance [9,10]. In this study, the material-related properties, including W_SP_, W_SL_ and viscosity of experimental composites containing EgGMA monomer as the target variable were the limiting factors and indicated EgGMA-quantity dependency as well. However, regardless of the other practical factors which may affect the composite performance, the viscosity and W_SP_ were visibly improved with EgGMA increase while DC and W_SL_ enhancement were limited to low EgGMA quantity. These results are consistent with the recently reported properties of such composites [10].

## 4. Conclusions

In this study, a eugenol-glycidyl methacrylate (EgGMA) monomer was incorporated into experimental dental composites, at 2.5 and 5 wt% (referred to as TBEg0 (control), TBEg2.5, and TBEg5). The rheological test indicates enhanced handling properties, which are better at higher temperatures (i.e., at 37 °C, compared with at 25 °C) with viscoelastic thinning behavior at higher shear forces. The degree of conversion (DC) and the water solubility (W_SL_) were slightly affected, whereas the water sorption (W_SP_) was enhanced compared with the reference. However, a significant effect on these variables was observed for TBEg5, compared with that of TBEg2.5, which proved that incorporating a small amount of EgGMA is favorable and highly ensures the desirable properties of the composites. Therefore, it could be concluded that the incorporation of EgGMA into dental composites was promising and deserves further in-depth investigations.

## Figures and Tables

**Table 1:** Compositions of the experimental composites.

Composite	Resins (wt%)			Fillers (wt%)		Initiation System (wt%, with Respect to the Total Monomers)	
	TEGDMA	BisGMA	EgGMA	S-HA	S-ZrO_2_	Initiator, CQ	Co-Initiator, DMAEMA
TBEg0	17.50	17.50	0.00	27.86	37.14	0.5	1.0
TBEg2.5	16.25	16.25	2.50	27.86	37.14	0.5	1.0
TBEg5	15.00	15.00	5.00	27.86	37.14	0.5	1.0

Abbreviations: BisGMA, bisphenol A-glycidyl methacrylate; CQ, camphorquinone; DMAEMA, 2-(*N*,*N*-dimethyl amino) ethyl methacrylate; EgGMA, eugenol-glycidyl methacrylate; S-HA, silanized hydroxyapatite; TEGDMA, triethylene glycol dimethacrylate; S-ZrO_2_, silanized zirconium dioxide.

**Table 2:** Degree of conversion (DC), complex viscosity (η*, kPa·s), Water sorption (W_SP_, wt%), and solubility (W_SL_, wt%) of TBEg composites. Standard deviations (SD) are given in brackets.

Composite	DC %; *n* = 3	Complex Viscosity η*, (kPa·s) at ω = 1.0 (rad/s); *n* = 3		W_SP_ (wt%); *n* = 5			W_SL_ (wt%); *n* = 5		
		25 °C	37 °C	1st Cycle	2nd Cycle	3rd Cycle	1st Cycle	2nd Cycle	3rd Cycle
TBEg0	68.75 ^a^ (0.750)	234.70 ^A,a^ (13.40)	52.01 ^B,a^ (9.64)	1.837 ^A,a^ (0.172)	2.975 ^B,a^ (0.113)	3.072 ^BC,a^ (0.050)	1.505 ^A,a^ (0.174)	0.072 ^B,a^ (0.051)	0.018 ^BC,a^ (0.005)
TBEg2.5	67.17 ^a^ (1.250)	86.44 ^A,b^ (16.47)	51.81 ^B,a^ (8.71)	1.819 ^A,a^ (0.153)	3.081 ^B,a^ (0.172)	2.933 ^BC,a^ (0.134)	1.590 ^A,a^ (0.227)	0.017 ^B,b^ (0.011)	0.042 ^BC,a^ (0.043)
TBEg5	58.01 ^b^ (1.018)	72.34 ^A,bc^ (17.14)	30.59 ^B,b^ (0.09)	1.409 ^A,b^ (0.189)	3.013 ^B,a^ (0.107)	2.728 ^C,a^ (0.121)	1.982 ^A,b^ (0.079)	0.009 ^Bb,c^ (0.009)	0.006 ^BC,a^ (0.027)

Note: Within each column, the different lowercase letters (^a, b, c^) indicate significant differences at *p* < 0.05. For each property (e.g., complex viscosity-at 25 and 37 °C; W_SP_-3 cycles; W_SL_-3 cycles) and within the same raw, the different uppercase letters (^A, B, C^) assigned to significant differences at *p* < 0.05.

## Data Availability

Data that support the findings of this study are included in the article.
